# Utility of neutrophil–lymphocyte ratio and platelet–lymphocyte ratio in predicting acute-on-chronic liver failure survival

**DOI:** 10.1515/biol-2022-0644

**Published:** 2023-07-15

**Authors:** Dong Li, Wei Sun, Li Chen, Jing Gu, Huichun Wu, Huayu Xu, Jianhe Gan

**Affiliations:** Department of Infection, The First Affiliated Hospital of Soochow University, No. 188, Shizi Street, Suzhou, Jiangsu, China

**Keywords:** acute-on-chronic liver failure, neutrophil–lymphocyte ratio, monocyte–lymphocyte ratio

## Abstract

This study explored the predictive value of the monocyte-to-lymphocyte ratio (MLR) and platelet–lymphocyte ratio (PLR) in patients with acute-on-chronic liver failure (ACLF). A retrospective analysis was carried out on 40 patients with ACLF from January 2018 and August 2019 in our hospital. The patient’s clinical information during hospitalization was collected, and their survivals were followed for 3 months. MLR and PLR values of patients were compared, and the correlation between liver function indicators and prognosis was analyzed. We observed that MLR levels in the survival and death groups were 0.521 (0.311, 0.827) and 0.741 (0.442, 1.121), respectively. MLR levels were markedly enhanced in the death group compared to the survival group (*P* = 0.021). The receiver operating characteristic curve (ROC) exhibited that the area under the ROC curve and 95% confidence interval for the survival group was 0.641 (0.528–0.757). Survival analysis demonstrated that the 3-month survival of the high MLR group was markedly lower than that of the low MLR group (*P* = 0.001). Multivariate regression exposed that MLR and PLR were independent prognostic factors for ACLF. MLR and PLR could be prospective prognosticative markers of ACLF.

## 1 Introduction

Liver failure (LF) is described as severe liver insufficiency and clinical complications caused by various factors according to the liver’s histopathological characteristics and disease progression rate [1]. LF could be separated into acute liver failure (ALF), sub-acute liver failure, acute-on-chronic liver failure (ACLF), and chronic liver failure [2]. The most common type in China is ACLF [3]. ACLF is an acute decompensation of liver function based on chronic liver disease, accompanied by abnormal blood coagulation and hyperbilirubinemia, ascites, hepatic encephalopathy, infection, and other complications [4]. ACLF is a disorder with speedy progress and high mortality [5]. Therefore, early evaluation of the prediction and effective treatment of ACLF patients is critical to promote the progress and prediction of ACLF patients.

Evidence has indicated that the progress and prognosis of ACLF are closely related to excessive systemic inflammation [6]. Hepatocyte necrosis releases various inflammatory factors and activates the immune response in the body, resulting in the migration of many granulocytes from bone marrow to peripheral blood [7,8]. There is evidence that the progress and prognosis of ACLF are closely related to excessive systemic inflammation [6]. It is a consensus that inflammatory cells and their ratios, such as platelets, neutrophils, lymphocytes, monocytes, neutrophils/lymphocytes (NLR) ratio, and platelet/lymphocyte (PLR) ratio play critical roles in the onset and progress of various diseases [9–11]. Severe liver inflammation characterized by neutrophils and lymphocytes is a vital factor affecting the onset and progress of liver failure [12]. In addition, platelets play a crucial role in hemostasis and inflammation. Mean platelet volume is a computerized measurement of mean platelet size and has been considered an inflammatory marker in many diseases [13]. Clinically, more and more evidence indicates that the combination of platelet and average platelet volume has greater clinical significance than using platelet or average platelet volume alone [14]. Recently, studies have shown that NLR is intently connected to the short-term prognosis of ALF [15]. However, the role of the PLR combined with NLR in predicting the survival of ACLF remains unknown and further studies are required. This study aimed to investigate whether PLR combined with NLR can predict the prognosis of patients with ACLF.

## 2 Methods

### 2.1 Analysis purpose and participants

During the study stage, 73 patients with ACLF treated in our hospital met the inclusion criteria between January 2018 and August 2019. Among them, 33 cases have incomplete clinical data. Finally, 40 participants were involved in this study. General clinical information and lab indicators, including age, albumin, neutrophil count, lymphocyte count, monocyte count, monocyte-to-lymphocyte ratio (MLR), NLR, alanine aminotransferase (ALT), aspartate aminotransferase (AST), prothrombin time, international standardized ratio (INR), and total bilirubin (TBil) were collected, and all patients were followed for at least 90 days through outpatient and inpatient systems and by telephone to assess short-term mortality. The participants have been selected according to the following inclusion criteria: ACLF patients diagnosed according to the “*Guidelines for Diagnosis and Treatment of Liver Failure (2018 Edition),*” defined as the short-term clinical manifestations of acute liver decompensation and LF based on chronic liver disease. The diagnostic criteria were as follows: rapid aggravation of jaundice, total serum bilirubin (TBil) ≥10× normal upper limit or daily increase of ≥17.1 µmol/L; signs of bleeding, INR ≥1.5. The exclusion criteria were as follows: age >80, patients with tumors and severe underlying diseases, patients with other viral hepatitis, or other liver diseases. The patients received routine comprehensive treatment, including liver protection, antivirus, albumin supplement, plasma and clotting substances, and other supportive treatment.

### 2.2 Ethical and legal considerations

This study was approved by the Institutional Ethics Review Committee of our hospital. This study was conducted based on the ethical standards of the Declaration of Helsinki of the World Medical Association. All participants signed the informed consent form.


**Informed consent:** Informed consent has been obtained from all individuals included in this study.
**Ethical approval:** The research related to human use has been complied with all the relevant national regulations, institutional policies and in accordance with the tenets of the Helsinki Declaration, and has been approved by Institutional Ethics Review Committee of First Affiliated Hospital of Soochow University.

### 2.3 Patient follow-up

According to the clinical data of the end point of treatment and 3 months after discharge, the patients were alienated into survival and death groups. The end point of treatment was the patients who died of ineffective drug treatment during hospitalization, gave up treatment when the condition worsened, and the treatment effect was converted to surgical liver transplantation.

### 2.4 Observation indicators

The fasting venous blood samples were collected from patients with ACLF 24 h after permission. The related markers were collected and analyzed, including white blood cell, neutrophil, lymphocyte, NLR, platelet, ALT, AST, TBil, cholinesterase, prothrombin activity, and INR. NLR, LMR, and PLR were calculated according to neutrophil, lymphocyte, monocyte, and platelet count. The liver and renal functions of the patients were detected by Shenzhen Mindray BS-400 automatic biochemical analyzer, INR was detected by compact automatic hemagglutination analyzer made by BE Company, and routine blood test was detected by automatic blood cell analyzer.

### 2.5 Statistical analysis

SPSS 22.0 and Prism 8 were used to analyze the data, and paired *T*-test was used to compare groups. The univariate analysis was applied to contrast the continuous variables of normal distribution, the Mann–Whitney *U*-test was employed to contrast the continuous data of non-normal distribution, and the Pearson chi-square test was employed to contrast the counting variables. The homogeneity of variance test was performed using Levene’s test. Pearson correlation analysis assessed the connection among the indicators; the binary normality test was performed before the Pearson correlation analysis. Kaplan–Meier was used to analyze the short-term death of subjects with high PLR + NLR and low PLR + NLR, and the ROC was employed to assess the predictive assessment of PLR + NLR of the outcome of ACLF patients. The ROC curve evaluated the value of diagnosis and prognosis, and the assessment level was *α* = 0.05; while for *P* < 0.05, the difference was statistically significant.

## 3 Results

### 3.1 Demographic characteristics of the research objects

**Figure 1:**
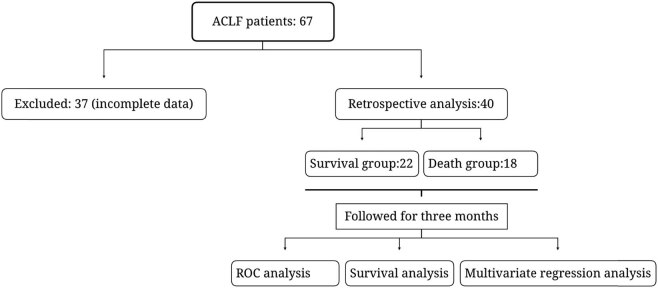
Clinical research flow diagram.

During the study period, 67 patients met the inclusion criteria. Among them, 37 patients were excluded due to the incomplete clinical medical record information. Finally, 40 patients with ACLF were enrolled in this retrospective study (Figure 1) including 31 males and nine females aged 31–79, with a median age of 51. The patients were alienated into survival and death groups according to the survival status of patients within 3 months. The 90-day survival of the patients was 55% (22/40) and the fatality rate was 45% (18/40). The NLR in the survival group was 3.23 (51.865, 5.358), which was markedly lesser than that in the death group (5.70, 52.878, 9.565), and the MLR in the survival group was 0.521 (0.0.311, 0.527), which was markedly lesser than that in the death group (0.0.440, 1.120) (Table 1).

**Table 1:** Difference in demographic characteristics between the death and survival groups

Variables	Survival group (*n* = 22)	Death group (*n* = 18)	*P*
Age (years)	45.580 ± 12.449	53.020 ± 11.679	0.001
Albumin (g/L)	32.544 ± 4.718	29.013 ± 4.417	0.001
Neutrophil count (10^9^/L)	3.870 (2.890, 5.220)	5.290 (3.428, 7.405)	0.015
Lymphocyte count (10^9^/L)	1.175 (0.728, 1.863)	0.930 (0.610, 1.698)	0.232
Monocyte count (10^9^/L)	0.605 (0.463, 1.028)	0.755 (0.433, 1.050)	0.027
MLR	0.521 (0.311, 0.827)	0.741 (0.442, 1.121)	0.021
NLR	3.2 (1.9, 5.4)	5.705 (2.878, 9.565)	0.004
ALT (U/L)	389.5 (143.6, 179.4)	242.0 (73.3, 733.5)	0.178
AST (U/L)	298.9 (118.7, 939.0)	290.4 (142.3, 594.2)	0.876
Prothrombin time (s)	20.0 (18.2, 23.2)	22.4 (18.6, 28.8)	0.034
INR	1.8 (1.6, 2.3)	2.0 (1.6, 2.6)	0.042
TBil (µmol/L)	264.0 (157.9, 378.5)	339.3 (168.5, 500.1)	0.162
MELD	15.2 (13.0, 18.3)	19.3 (14.6, 25.1)	0.003

MLR: monocyte–lymphocyte ratio; NLR: neutrophil–lymphoid cell ratio; ALT, alanine aminotransferase; AST, aspartate aminotransferase; MELD, end-stage liver disease model score.

### 3.2 Correlation between MLR and liver function indicators

Pearson correlation analysis showed that NLR and MLR did not correlate with TBil, ALT, or AST. In contrast, they had a positive correlation with INR and PT and a negative correlation with albumin (Table 2).

**Table 2:** Correlation analysis of leukocyte ratio and liver function indicators

Indicators		ALT	AST	TBil	INR	PT	Alb
NLR	*r*	−0.105	−0.115	0.071	0.250	0.254	−0.350
	*P*	0.318	0.276	0.502	0.016	0.015	0.001
MLR	*r*	−0.168	−0.154	0.126	0.208	0.206	−0.312
	*P*	0.109	0.143	0.231	0.046	0.049	0.002

MLR, monocyte–lymphocyte ratio; NLR, neutrophil–lymphoid cell ratio; ALT, alanine aminotransferase; AST, aspartate aminotransferase; TBiL, total bilirubin; INR, international standardized ratio; PT, prothrombin time; Alb, albumin.

### 3.3 Correlation analysis between MLR and forecast of patients with ACLF

After adjusting for other factors, the multivariate analysis showed that NLR and MLR were independent prognostic factors in HBV-ACLF subjects (Table 3). There was no significant correlation between infection rate and NLR and MLR at baseline (*P* = 0.005 and 0.026).

**Table 3:** Association analysis of NLR and MLR and prognosis

Indicators	*β*	SE	Wald *χ* ^2^	OR	95% CI	*P*
NLR	0.151	0.054	7.804	1.162	1.046–1.292	0.005
MLR	0.550	1.220	4.928	3.387	1.154–9.944	0.026

MLR: monocyte–lymphocyte ratio; NLR, neutrophil–lymphocyte ratio; CI, confidence interval; SE, standard error; OR: odds ratio.

### 3.4 The value of MLR in forecasting the prognosis of patients

**Figure 2:**
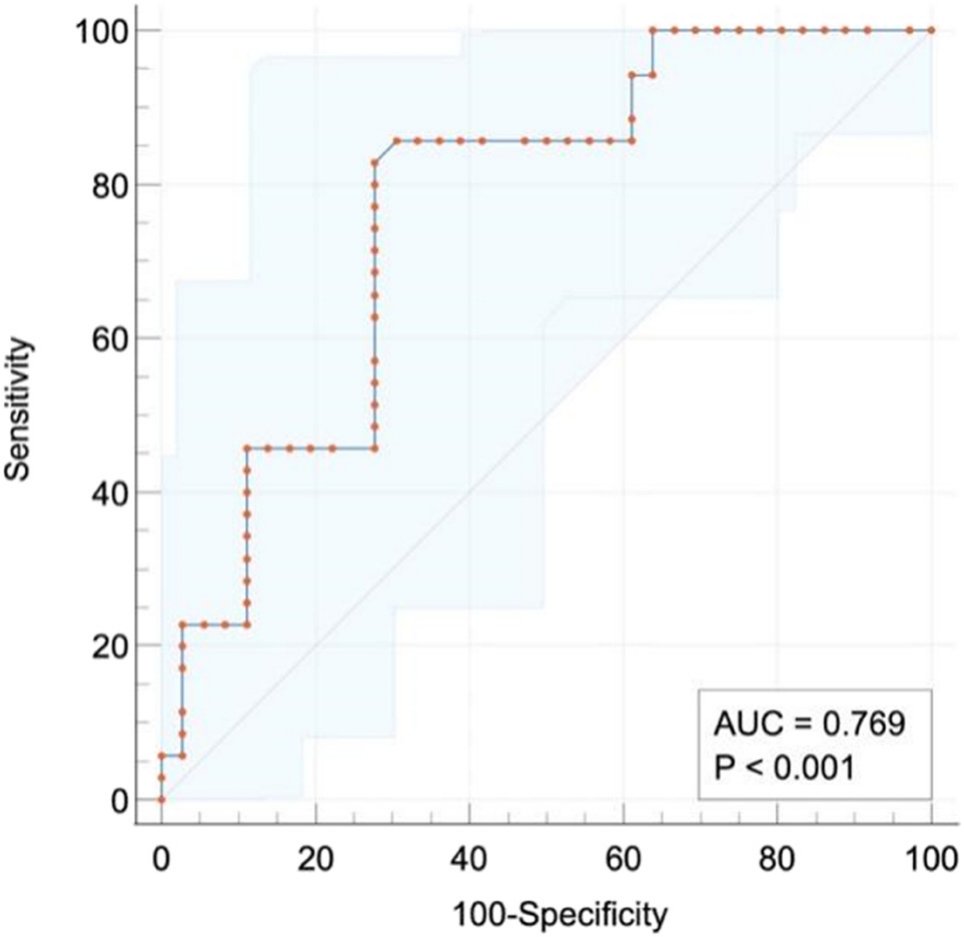
ROC of serum NLR, MLR, and MELD for forecasting the prognosis of ACLF.

**Figure 3:**
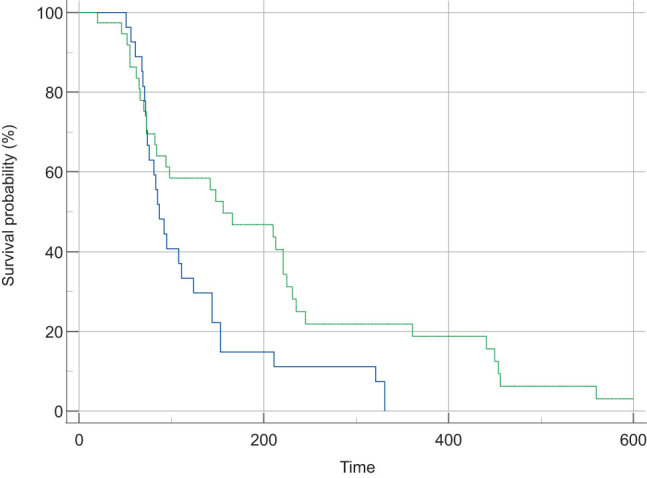
Comparison of survival rate of non-transplantation in different NLR and MLR groups.

ROC curve exposed that the area under the curve (AUC) of NLR to forecast the 90-day survival rate of ACLF patients was 0.67 (50.565–0.786). When NLR was 5.957, the sensitivity and specificity were 0.500 and 0.833, respectively. The AUC of MLR to predict the 90-day survival rate of ACLF patients was 0.641 (0.528–0.757). When MLR was 0.398, the sensitivity and specificity were 0.865 and 0.398, respectively. The AUC of the model for end-stage liver disease (MELD) score for predicting the 90-day survival rate in patients with ACLF was 0.679 (0.567–0.792). When MELD was 19.740, the sensitivity was 0.500, and the specificity was 0.896 (Figure 2). There were 62 cases in the low NLR group with NLR < 5.957. Survival analysis exhibited that the 90-day survival in the high NLR group (*P* = 0.0005) was markedly decreased compared with that in the corresponding low NLR group (Figure 2). The cut-off value of MLR was 0.398, with MLR ≥0.398 as the high MLR group (*n* = 67) and MLR <0.398 as the low MLR group (*n* = 25). Survival analysis exhibited that the 90-day survival of the high MLR group was markedly decreased compared with that of the low MLR group (Figure 3). Therefore, MLR ≥0.398 is an independent prognosticator of short-term survival in patients with ACLF.

## 4 Discussion

ACLF is a multifaceted clinical disease with elevated mortality, and LF is the main pathological change [16]. As reported previously, liver cirrhosis gradually develops into end-stage or non-end-stage liver failure, with liver synthesis dysfunction as the main pathological change, characterized by lack of various coagulation factors, serum albumin and α β globulin, an increase of bilirubin and so on [17]. It seriously affects the liver’s synthesis, secretion, detoxification function, and the poor prognosis of ACLF. Currently, there is no effective treatment for ACLF. Emerging evidence suggests that the serum levels of TNF-α, IL-2, IL-6, IL-8, and INF-γ in patients with ACLF were markedly higher than those in healthy volunteers. In contrast, the expression of T lymphocytes (CD4+ T lymphocytes, CD8+ T lymphocytes), natural killer cells, and natural killer T cells was significantly decreased, suggesting that inflammation is involved in the pathogenesis of ACLF [18]. Horvatits et al. indicate that high levels of NLR are positively correlated with liver lesions such as hepatic lobular injury, hepatic fibrosis, fatty liver, and so on. A recent study has highlighted that immune system diseases are intently connected to the high level of NLR expression [19].

In addition, NLR could effectively predict systemic inflammation and reflect the dynamic variations of liver damage to some extent. By comparing the number of lymphocytes and neutrophils in the serum of ACLF and healthy volunteers, it is concluded that the lessening of serum NLR in patients with ACLF implies the occurrence of inflammation, can predict the severity of disease progression, and is of great significance in directing clinical therapy [8]. A growing body of evidence has indicated that systemic inflammation usually occurs in patients with ACLF, which may be connected to poor prognosis. In recent years, some studies have analyzed the roles of systemic inflammation markers in ACLF and found that the content of inflammation markers in patients with ACLF were significantly increased [20]. However, our understanding of neutrophil–lymphocyte ratio and platelet–lymphocyte ratio in predicting ACLF survival remains insufficient. To the best of our knowledge, this is the first validation of NLR and PLR in predicting the survival of patient with ACLF.

Previous studies have demonstrated that the age difference between survivors and deaths cannot be ignored [21]. The physical function of elderly patients is weakened, and the number of functional hepatocytes might be lower than that of young people, so this study excluded patients over 80. In this study, we found that the significant increase in MLR in the death group was mainly due to the decrease in the number of lymphocytes and the rise in the number of monocytes. We also observed that the level of lymphocytes in the dead group showed a downward trend compared with the survival group, but there was no statistical significance. This decline can be attributed to lymphocytopenia [22]. Inflammatory stimulation mainly affects the number of monocytes in the blood of patients with ACLF, which leads to a change in MLR [23]. Patients with ACLF have abnormal NLR and MLR, and their levels are associated with coagulation indicators such as INR, PT, and albumin. In contrast, the levels of MLR and NLR could reflect the extent of inflammation and prognosis of ACLF to some extent [24]. Low lymphocyte count could indicate malnutrition and an inflammatory state. High monocyte and low lymphocyte amount can also suggest the severity and development of liver damage in patients with ACLF [25]. In this study, an increase in MLR was observed in dead patients, indicating that inflammation was continuing, which might lead to a poor prognosis. MELD scores are often employed to forecast the viability of end-stage liver disease patients and assess the necessity of liver transplantation. A previous study showed that high levels of NLR were closely correlated with pathological liver changes such as hepatic lobular injury, hepatic fibrosis, and fatty liver. In addition, NLR can effectively predict systemic inflammation and reflect the dynamic changes of liver damage to some extent [26]. However, the application of the utility of neutrophil–lymphocyte ratio and platelet–lymphocyte ratio in predicting ACLF survival remains unknown. The results of this study suggested that MELD score, MLR, and NLR were independent indicators for predicting the 90-day survival of patients with ACLF. Moreover, the predictive ability of the MELD score (AUC = 0.679) was enhanced than that of MLR (AUC = 0.641) and NLR (AUC = 0.675). MLR and NLR need only two indicators to calculate, which is simpler and more convenient than MELD. However, the present study also has some limitations. Only a few cases were included in this study. Subsequently, the sample size can be expanded, and subgroup analysis can be carried out further to assess the application of the utility of neutrophil–lymphocyte ratio and platelet–lymphocyte ratio in predicting ACLF survival.

In conclusion, we validated that NLR and MLR are related to the prognosis of ACLF and could be employed as potential markers to evaluate the prognosis of patients with ACLF. The leukocyte ratio could be considered an assessment means for early managing ACLF patients during hospitalization.
